# Comparison of clinical outcomes of arthroscopic rotator cuff repair utilizing suture-bridge procedures with or without medial knots: a meta-analysis

**DOI:** 10.1186/s12893-023-02060-0

**Published:** 2023-06-13

**Authors:** Qiu Huang, Xiaoyu Li, Ye Zhang, Changchun Jian, Hai Mou, Yunsheng Ou

**Affiliations:** 1 Department of Orthopedics, People’s Hospital of Leshan, Shizhong District, Leshan, Sichuan China; 2 Department of Orthopedics, The First Affiliated Hospital of Chongqing Medical University, Chongqing, China; 3 Humanities and Management college, Southwest Medical University, Longmatan District, Luzhou, Sichuan China

**Keywords:** Rotator cuff repair, Suture bridge, Knotted, Knotless, Meta-analysis

## Abstract

**Purpose:**

This investigation aimed to compare the medical efficacy of the knotted and knotless suture-bridge procedures in rotator cuff repair.

**Methods:**

The Pubmed, Embase, and Cochrane Library datasets were searched for all available publications comparing the medical results of arthroscopic rotator cuff repairs utilizing knotted or knotless suture-bridge procedures. Two researchers utilized Newcastle-Ottawa Scale and Cochrane risk-of-bias tool to evaluate the included studies. Employing Revman 5.3 software, meta-analysis was conducted following the PRISMA reporting guideline.

**Results:**

Eleven investigations with 1083 patients were considered suitable for the final meta-analysis. 522 individuals were assigned to the knotted group, whereas 561 were assigned to the knotless group. No statistical difference was found between the knotted and knotless groups, regarding VAS score (WMD, 0.17; 95% CI, − 0.10 to 0.44; P = 0.21); Constant score (WMD, -1.50; 95% CI, − 3.52 to 0.52; P = 0.14); American Shoulder and Elbow Surgeons Shoulder (WMD, -2.02; 95% CI, − 4.53 to 0.49; P = 0.11); University of California Los Angeles score (WMD, -0.13; 95% CI, − 0.89 to 0.63; P = 0.73); ROM of flexion (WMD, 1.57; 95% CI, − 2.11 to 5.60; P = 0.37), abduction (WMD, 1.08; 95% CI, − 4.53 to 6.70; P = 0.71) and external rotation (WMD, 1.90; 95% CI, − 1.36 to 5.16; P = 0.25); re-tear rate (OR, 0.74; 95% CI, 0.50 to 1.08; P = 0.12), and medical complications (OR, 0.90; 95% CI, 0.37 to 2.20; P = 0.82).

**Conclusion:**

For arthroscopic rotator cuff repairs, there were no statistical differences in medical results among knotted and knotless suture-bridge procedures. Overall, both techniques showed excellent clinical outcomes and could be safely utilized to treat rotator cuff injuries.

**Supplementary Information:**

The online version contains supplementary material available at 10.1186/s12893-023-02060-0.

## Introduction

The conventional suture bridge techniques involved medial knots in the medial row of the tendon to provide increased repair strength and less gap development [1, 2]. However, the medial knots may cause strangulation of the repaired tendon and eventually hamper the healing process and raise the chance of type 2 re-tears [3–5]. To avoid these risks, the knotless suture bridge techniques were implemented, in which the sutures were attached to medial anchors, passed through the tendon without knots, then secured with lateral knotless anchors [6, 7]. At present, both techniques are widely used in treating rotator cuff tears.

As biomechanical studies reported, knotted suture bridge techniques tended to have superior biomechanical properties, including greater maximum load, higher failure stiffness and less gap development than knotless suture bridge techniques [8]. However, the conclusions from biomechanical studies may not correlate to clinical outcomes directly. Additionally, the effects of knotless technique in promoting healing process and preventing re-tears are also uncertain. Despite the publication of a number of studies contrasting the medical results of knotted and knotless suture bridging procedures, the medical efficacy of the two procedures remains controversial. The comprehensive review performed by Elbuluk et al. documented that the knotted and knotless suture bridge techniques significantly improved functional consequences following rotator cuff repairs, and the failure rates in the knotless group showed a downtrend [9]. Unfortunately, the study above lacked meta-analysis, and most of included studies did not contrast the postoperative results among the two procedures directly. Following a review of recently published articles, a number of new studies comparing the medical results of the two procedures were retrieved. Therefore, an updated meta-analysis was required to make the conclusion more convincing. This study presents an report on an updated meta-analysis incorporating newly published studies that directly compare the postoperative outcomes between suture bridge technique with knots or not.

## Materials and methods

### Criteria for inclusion and exclusion

Criteria for inclusion: (1) clinical studies reporting arthroscopic rotator cuff repairs utilizing suture-bridge procedures; (2) investigations that directly compare the postoperative results of knotted and knotless suture-bridge procedures; (3) at least one of the following outcomes was revealed: pain relief, functional scores, re-tear rate, range of motion (ROM), and the occurrence of complications. Criteria for exclusion: (1) cadaveric research, animal studies, case reports, and reviews; (2) unable to get the entire text; or (3) insufficient original information.

### Search strategy

From their establishment through April 11, 2022, the databases PubMed, Embase, and the Cochrane Library were searched. The search was carried out utilizing the following algorithm: (“rotator cuff” OR “supraspinatus” OR “infraspinatus” OR “subscapularis” OR “teres minor”) AND (“suture bridge” OR “double row”) AND (“knot” OR “knotted” OR “knotless”).

The findings were imported into the Endnote program, and duplication were eliminated. Two authors independently reviewed the titles and abstracts to omit papers that didn’t match the eligibility requirements. Thereafter, the complete texts of the possibly included investigations were accessed to select the final articles that were included. Additionally, the references of the selected research were reviewed to determine other potentially relevant papers. Discrepancies were handled by debate; In the absence of unanimity, a senior reviewer was tasked with making the ultimate decision.

#### Data extraction and quality assessment

Two independent reviewers analyzed the methodological quality of the selected papers employing the NOS (Newcastle-Ottawa Scale) and Cochrane risk of bias criteria. Differences of opinion were handled by debate; if consensus couldn’t be established, a senior reviewer was tasked with making the ultimate decision. Randomized controlled trials (RCT) were evaluated utilizing the Cochrane risk-of-bias guidelines, whilst the other investigations were evaluated using the NOS. According to the NOS criteria, the methodological quality of the investigations was scored regarding three domains: including choice of research cohorts, comparability of cohorts, and result ascertainment. The total score was 10 (range from 0 to 10), and studies with NOS scores ≥ 6 were considered with high-quality. While in Cochrane risk-of-bias criteria, the items of the trials, including randomization sequence generation, allocation concealment, blinding of people involved and personnel, blinding of outcome measures, incomplete outcome information, selective reporting, and other biases were assigned as low risk, high risk, or unknown risk.

#### Outcome measures

Data extraction, including study characteristics, participants’ demographic information, and clinic outcomes concerning pain relief, function-related scores, re-tear rate, ROM, and incidence of complications, was performed by two independent authors and proofread by a third one. The primary outcomes involved shoulder pain, shoulder function scales, and ROM. Pain evaluation was computed by visual analog scale (VAS), and ROM was assessed by the passive motion data of forward flexion, abduction and external rotation. The functional assessments were evaluated by Constant score (CS), American shoulder and elbow surgeons score (ASES), and University of California Los Angeles score (UCLA). The secondary outcomes included re-tear rate and incidence of complications. If outcome measures were evaluated at numerous time-points, data from the last time point were utilized in the meta-analysis.

### Statistical analysis

Employing Revman 5.3 software, Meta-analysis was conducted following the PRISMA(Preferred Reporting Items for Systematic Review and Meta-analyses) reporting guidelines[10]. The odds ratio (OR) was utilized to evaluate dichotomous events (re-tear rate and incidence of complications), and weighted mean difference (WMD) was utilized to evaluate continuous information (VAS, ROM, CS, ASES, UCLA) with a 95% confidence interval (CI). A P-value below 0.05 was regarded as statistical significance. The statistical heterogeneity was estimated utilizing Q and I^2^. Heterogeneity was regarded if I^2^ > 50% and P ≤ 0.1. When I^2^ < 50% and P > 0.1, the fixed-effect approach was employed; on the contrary, when I^2^ > 50% and P < 0.1, the random-effect approach was adopted, and the source of heterogeneity was analyzed by omitting studies one at a time to see the influence on the pooled outcomes. Forest plots were used to present the results. Utilizing the funnel plot of the most often documented result, publication bias was evaluated.

## Results

### Research selection

**Fig. 1:**
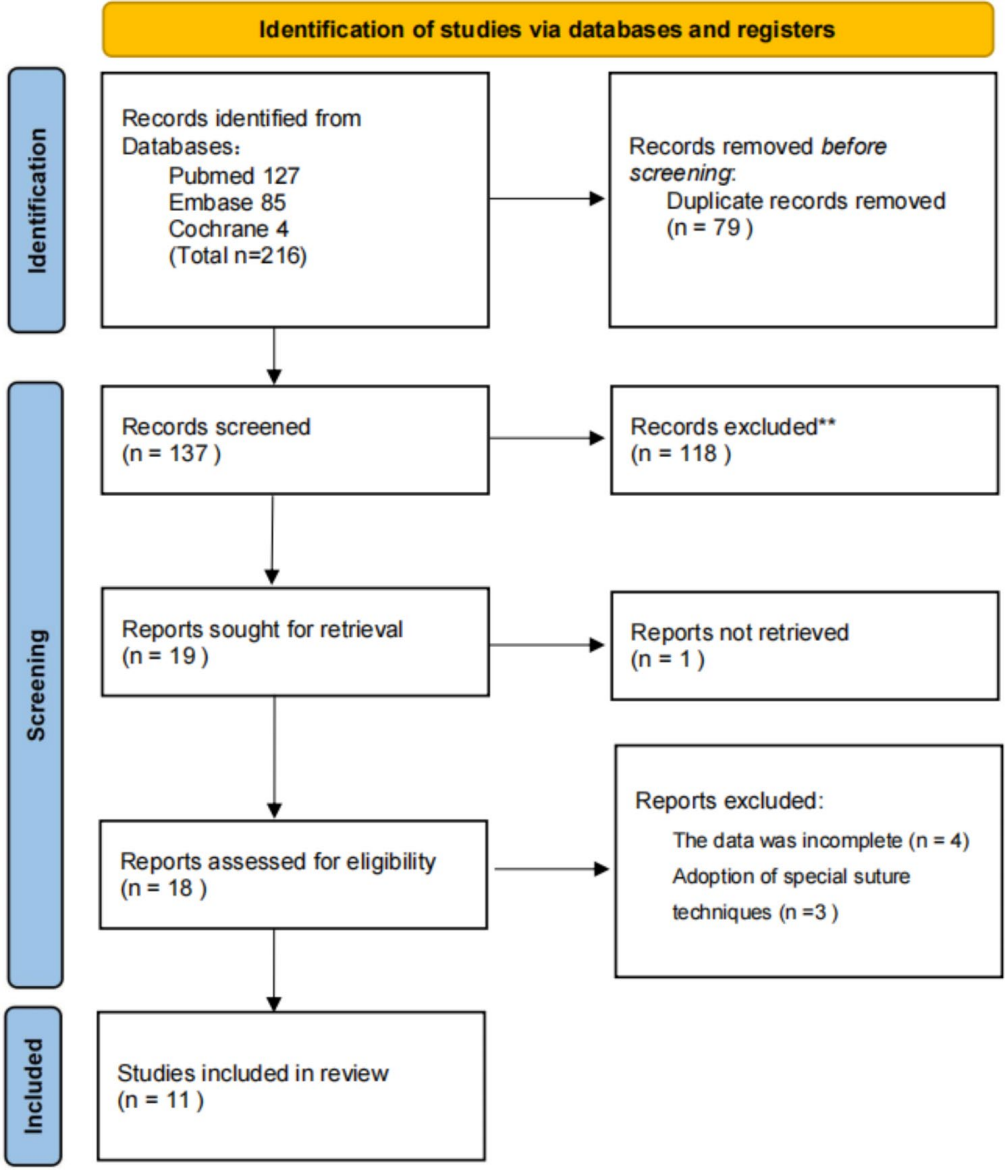
PRISMA flow diagram

As shown in the flow diagram (Fig. 1), the described search algorithm provided 216 findings (127 in Pubmed, 85 in Embase, and 4 in Cochrane Library), of which 79 publications were discarded due to duplication and 118 trials were deleted following reading the title or abstract. Eventually, by reading the full texts, one randomized controlled trial, nine high-quality retrospective studies with NOS scores ≥ 6, and one non-randomized prospective study with a NOS score of 8 matched the requirements for inclusion and were involved in the meta-analysis [2, 6, 11–19].

### Study characteristics

In total, there were 1083 patients involved in the eleven included trials. among them, 522 patients were managed with the knotted suture-bridge method (knotted group) and 561 patients with the knotless suture-bridge method (knotless group). Table 1 displays the features of the selected trials.

**Table 1:** Features of selected studies

Study	Design	Level of evidence	Total subjects	Male/ Female	Age (Year)	Duration of follow-up(mo)	Outcomes measured
Boyer et al. 2015 [11]	Prospective cohort study	3	73				VAS, CS, ROM, Re-tear rate
Knotted			38	22/16	58(47–72)	29(23–32)	
Kontless			35	21/14	59(44–68)	21(12–23)	
Burns et al. 2019 [12]	Retrospective study	4	37				VAS. SST, ASES, UCLA
Knotted			15	6/9	61.6 ± 9.1	30(28–30)	
Kontless			22	9/13	63.2 ± 9.7	13.5(11.5–15.5)	
Gürpınar et al. 2019 [2]	Retrospective study	NR	121				VAS, CS, ROM, Re-tear rate
Knotted			64	32/32	56.7 ± 7.7	19.3 ± 4.7	
Kontless			57	23/34	56.6 ± 7.0	18.7 ± 4.6	
Honda et al. 2018 [13]	Retrospective study	3	53				JOA, UCLA, Re-tear rate
Knotted			29	17/12	63.8 ± 8.4	24	
Kontless			24	15/9	65.1 ± 9.6	24	
Hug et al. 2015 [14]	Retrospective study	3	42				CS, SSV, WORC, Re-tear rate
Knotted			20	15/5	61.2 ± 7.5	23.4 ± 2.9	
Kontless			22	14/8	63.3 ± 7.2	24.4 ± 4.8	
Kim et al. 2014 [15]	Retrospective study	3	157			6.21(3–33)	Re-tear rate
Knotted			96	NR	NR	NR	
Kontless			61	NR	NR	NR	
Kim et al. 2018 [16]	Prospective cohort study	NR	100				VAS, CS, UCLA, ASES, Re-tear rate
Knotted			50	28/22	59.4 ± 7.45	24	
Kontless			50	24/26	59.9 ± 7.66	24	
Millett et al. 2017 [6]	Retrospective study	3	137	NR	59 ± 10	34.8(24-64.8)	ASES, SF-12 PCS
Knotted			35	NR	NR	NR	
Kontless			102	NR	NR	NR	
Pogorzelski et al. 2019 [17]	Retrospective study	3	192		60(23–80)	79.2(60–132)	ASES, SF-12 PCS, Quick DASH, SANE
Knotted			69	26/43	NR	NR	
Kontless			123	94/29	NR	NR	
Şahin et al. 2021 [18]	RCT	1	88				VAS, CS, ROM,Re-tear rate
Knotted			42	12/30	54.3 ± 9.8	25.4 ± 8.3	
Kontless			46	20/26	55.8 ± 8.2	23.3 ± 7.2	
Zwolak et al. 2022 [19]	Retrospective study	4	83				Quick DASH, SPADI, ROM, strength
Knotted			64	35/29	61(42–75)	12	
Kontless			19	11/8	65(52–81)	12	

VAS, Visual analog scale pain score;CS, Constant score; SST, Simple shoulder test; ASES, American shoulder and elbow surgeons score; UCLA, University of california los angeles score; ROM, Range of motion; JOA, Japanese Orthppaedic Association score; SSV, Subjective shoulder value; WORC, Western ontario rotator cuff score; SF-12 PCS, Short-Form 12 physicial component summary; Quick DASH, Quik Qisabilitied of the Arm, Shoulder and Hand score; Quick DASH, Quik Qisabilitied of the Arm, Shoulder and Hand score; SANE, Single Assessment Numeric Evaluation; SPADI, Shoulder Pain and Disability score; RCT, Randomized controlled trial; NR, No Report

### Evaluation of risk of bias

**Fig. 2:**
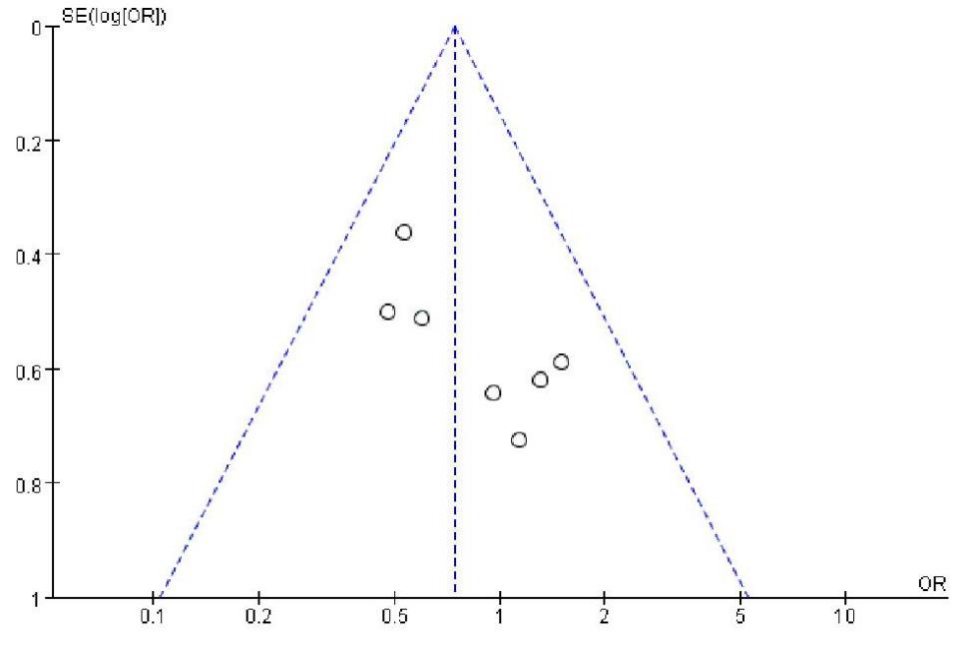
Funnel plot of the most reported outcome(re-tear)

One RCT and ten comparative trials were involved in this meta-analysis. Cochrane risk-of-bias criteria were adopted to the included RCT, with the following findings: randomization sequence generation: low risk; allocation concealment: high risk; blinding of participants and personnel: unclear; blinding of outcome measures: high risk; incomplete outcome information: low risk; selective reporting: low risk; and other bias: unclear. Other trials were evaluated using NOS (Table 2). The majority of studies’ retrospective design and absence of blinding raise the risk of selecting and detecting bias, which is the most important restriction of the current analysis. In the involved trials, the risks of attrition bias, reporting bias, and other kinds of bias were low. Given the high NOS scores (rang: 6–8) of the comparative trials, the overall bias of the investigations was moderate. The possibility of publication bias was investigated utilizing a funnel plot of the most often stated finding (re-tear). Regarding the center of distribution, the dispersion of the plots was good, indicating a low to moderate risk of publication bias. The detailed quality assessments for each retrospective studies and non-randomized prospective studies are summarized in Table 2, and the funnel plot is illustrated in Fig. 2.

**Table 2:** The Newcastle-Ottawa Scale (NOS) for evaluating the retrospective trials

Study	Selection	Comparability	Exposure	Total scores
Boyer et al. 2015 [11]	***	**	***	8
Burns et al. 2019 [12]	***	*	***	7
Gürpınar et al. 2019 [2]	***	**	**	7
Honda et al. 2018 [13]	***	**	***	8
Hug et al. 2015 [14]	***	*	**	6
Kim et al. 2014 [15]	***	**	**	7
Kim et al. 2018 [16]	***	**	**	7
Millett et al. 2017 [6]	***	**	**	7
Pogorzelski et al. 2019 [17]	***	**	**	7
Zwolak et al. 2022 [19]	***	**	**	7

### Meta-analysis results

#### Pain relief

**Fig. 3:**
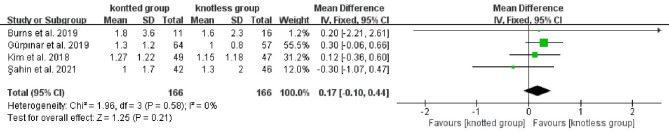
Forest plot for comparison of VAS

Four trials[2, 12, 16, 18] provided VAS scores, with 166 individuals in each (knotted or knotless) group. The heterogeneity analysis revealed non-heterogeneity ((P = 0.58, I^2^ = 0%), hence a fixed effect model (FEM) was utilized. Meta-analysis demonstrated no statistical difference among the two groups (WMD, 0.17; 95% CI, − 0.10 to 0.44; P = 0.21; Fig. 3). The pooled results of VAS score analyses were not significantly changed by omitting studies one at a time.

#### Functional improvement

**Fig. 4:**
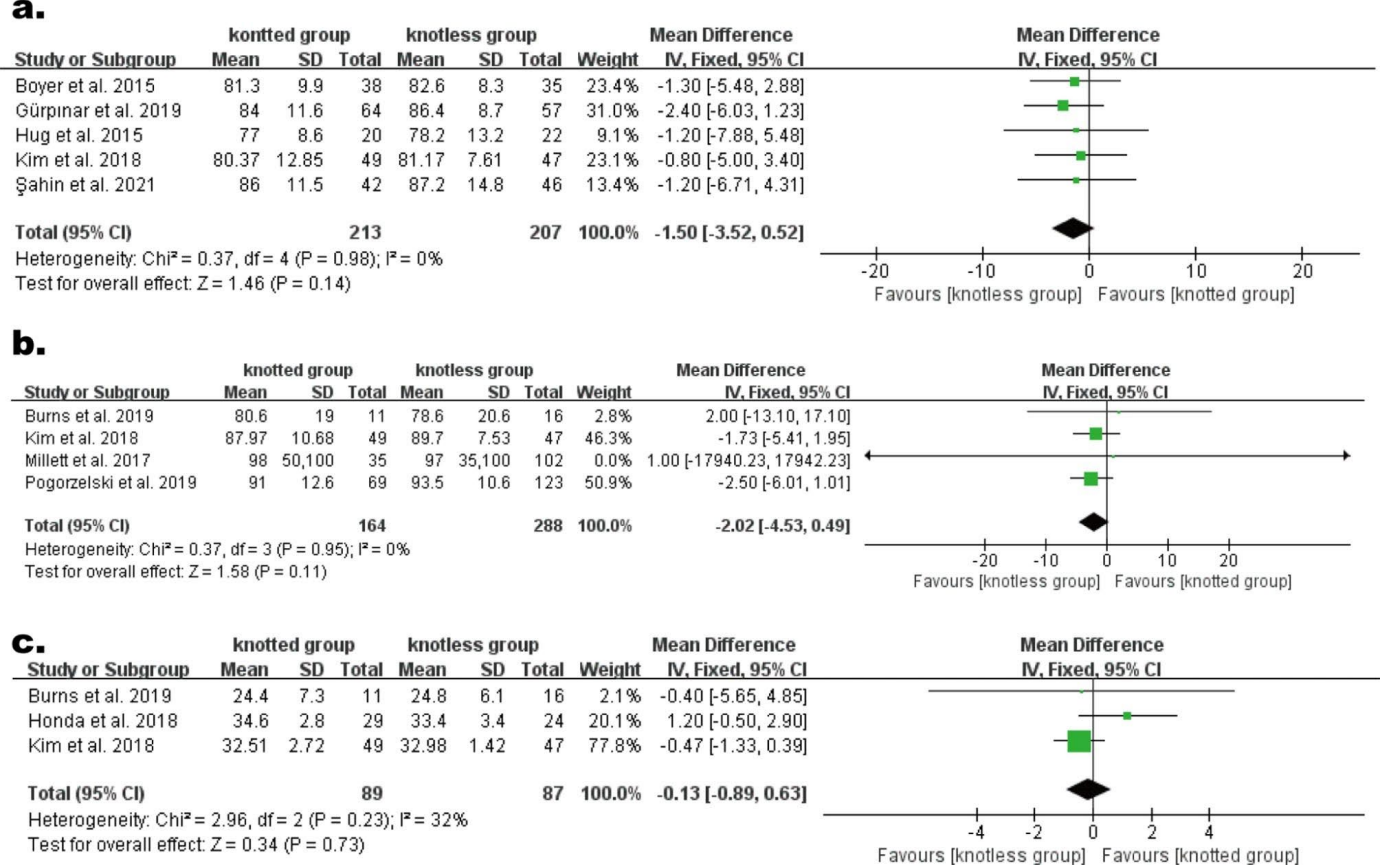
Forest plot for comparison of function-related scores. (**a**) Constant score, (**b**) ASES score, (**c**) UCLA score

The Constant score was reported in five trials[2, 11, 14, 16, 18], comprising 213 individuals in knotted group and 207 individuals in knotless group. The heterogeneity result demonstrated non-heterogeneity (P = 0.98, I^2^ = 0%), hence a FEM was employed. Meta-analysis demonstrated no statistical difference among the two groups (WMD, -1.50; 95% CI, − 3.52 to 0.52; P = 0.14; Fig. 4a).

The ASES score was reported in four studies [6, 12, 16, 17], comprising 164 individuals in knotted group and 288 individuals in knotless group. The heterogeneity result indicated no heterogeneity (P = 0.95, I^2^ = 0%), so a FEM was utilized. Meta-analysis demonstrated no statistical difference among the two groups (WMD, -2.02; 95% CI, − 4.53 to 0.49; P = 0.11; Fig. 4b).

The UCLA score was reported in three studies[12, 13, 16], comprising 89 individuals in knotted group and 87 individuals in the knotless group. The heterogeneity result indicated moderate heterogeneity (P = 0.23, I^2^ = 32%), so a FEM was utilized. Meta-analysis demonstratedno statistical difference among the two groups. (WMD, -0.13; 95% CI, − 0.89 to 0.63; P = 0.73; Fig. 4c).

The pooled findings of functional assessment analyses were not significantly changed by omitting studies one at a time.

#### ROM

**Fig. 5:**
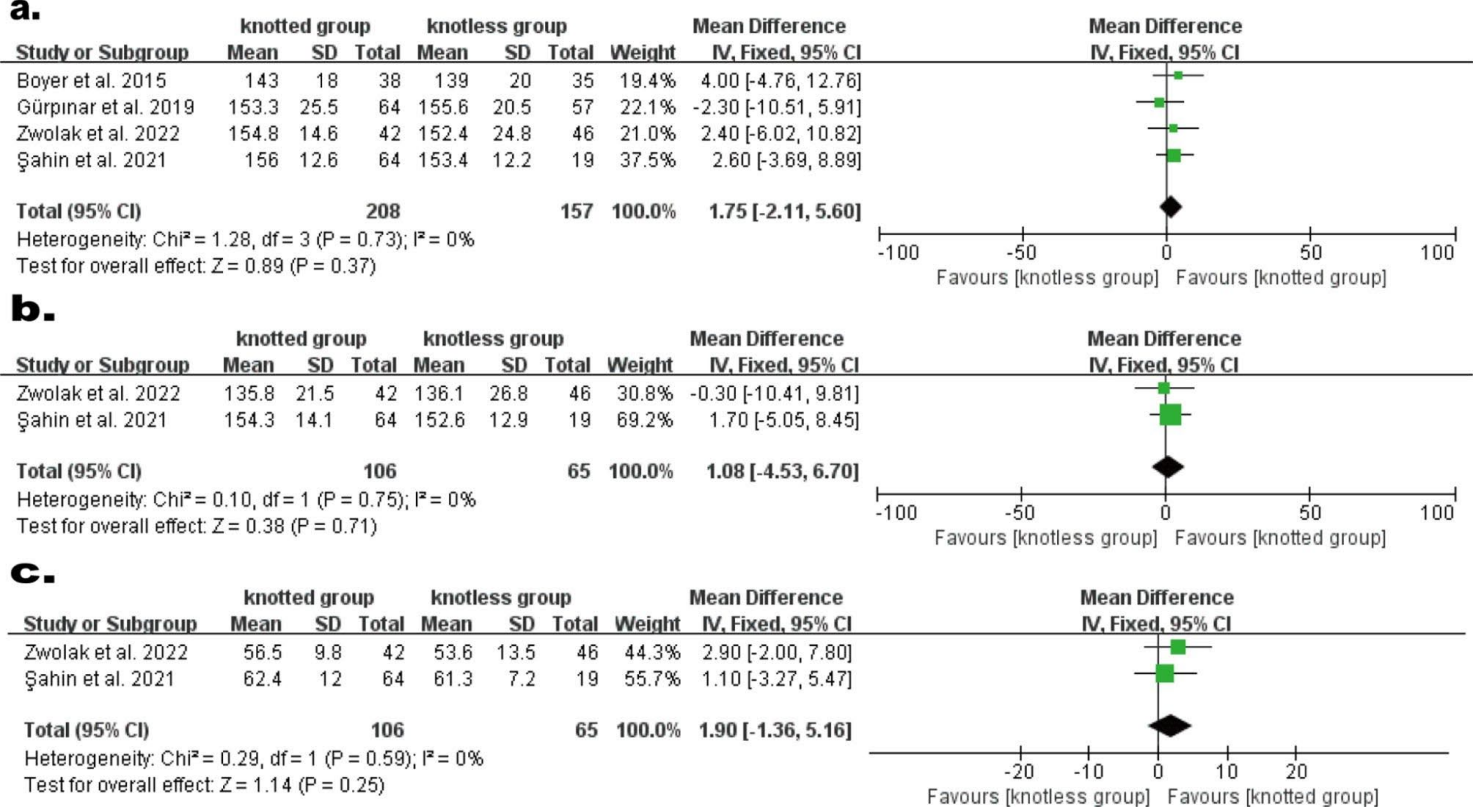
Forest plot for comparison of ROM. (**a**) forward flexion, (**b**) abduction, (**c**) external rotation

The ROM of forward flexion was reported in four studies [2, 11, 18, 19], comprising 208 individuals in knotted group and 157 patients in knotless group. The heterogeneity result indicated non- heterogeneity (P = 0.73, I^2^ = 0%), so a FEM was employed. Meta-analysis demonstrated no statistical difference among the two groups (WMD, 1.57; 95% CI, − 2.11 to 5.60; P = 0.37; Fig. 5a). The ROM of abduction and external rotation were reported in two studies comprising 106 individuals in knotted group and 65 individuals in the knotless group[18, 19]. The heterogeneity results of abduction (P = 0.75, I^2^ = 0%) and external rotation (P = 0.59, I^2^ = 0%) indicated no heterogeneity, so the FEM was utilized. Meta-analysis demonstrated no statistical difference among the two groups in terms of abduction (WMD, 1.08; 95% CI, − 4.53 to 6.70; P = 0.71; Fig. 5b) and external rotation (WMD, 1.90; 95% CI, − 1.36 to 5.16; P = 0.25;Fig. 5c). The pooled results of ROM analyses were not significantly changed by omitting studies one at a time.

#### Re-tear rate

**Fig. 6:**
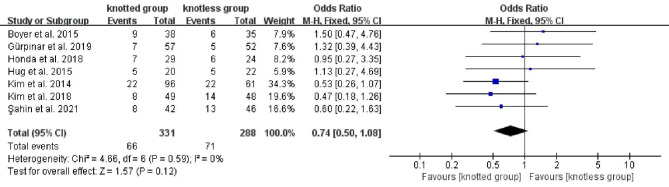
Forest plot for comparing the re-tear rate

Re-tear rate was documented in seven studies[2, 11, 13–16, 18], comprising 311 individuals in knotted group and 288 individuals in knotless group. The heterogeneity result indicated no heterogeneity (P = 0.59, I^2^ = 0%), so a FEM was utilized. Meta-analysis demonstrated no statistical difference among the two groups (OR, 0.74; 95% CI, 0.50 to 1.08; P = 0.12;Fig. 6). The pooled results of re-tear rate analyses were not significantly changed by omitting studies one at a time.

#### Medical complications

**Fig. 7:**
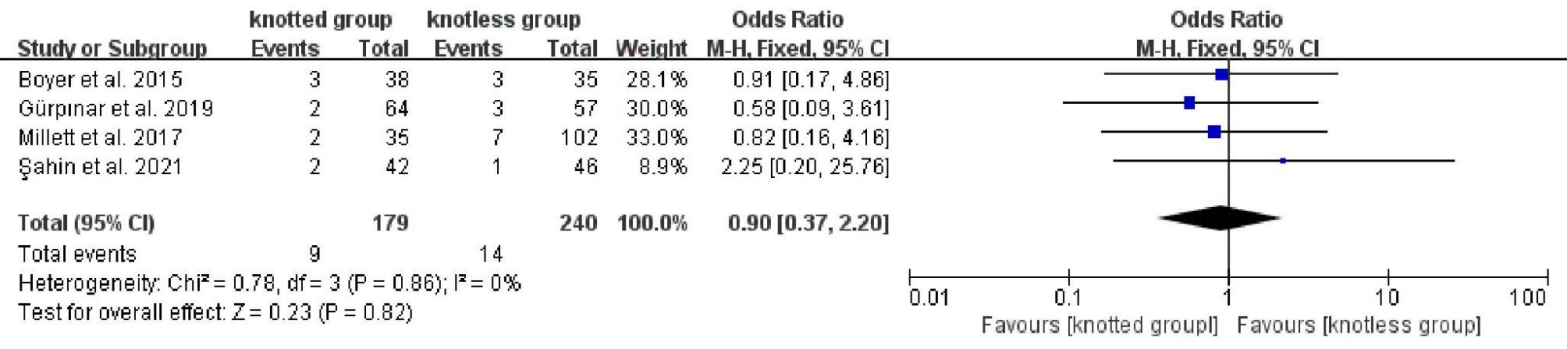
Forest plot for comparison of medical complications

The medical complications, including stiffness, infection, hematoma, ruptured biceps tenodesis, superficial fistula, and acute pain, were reported in four studies[2, 6, 11, 18], comprising 179 individuals in knotted group and 240 patients in knotless group. The heterogeneity result indicated no heterogeneity (P = 0.86, I^2^ = 0%), so a FEM was employed. Meta-analysis revealed that neither group differed significantly from the other (OR, 0.90; 95% CI, 0.37 to 2.20; P = 0.82; Fig. 7). The pooled results of complication analyses were not significantly changed by omitting studies one at a time.

## Discussion

This study collected trials comparing the clinical efficacy between the knotted and knotless suture bridge procedures for rotator cuff repairs and performed a meta-analysis. In regards of pain relief, postoperative function, and complications, neither the knotted nor the knotless suture bridging procedures demonstrated superiority over the other. The lack of a statistical difference in re-tear rates indicates that the knotless suture bridge procedure is unable to overcome the shortcomings of the knotted procedures.

Although many novel procedures for the arthroscopic repair of rotator cuff tears have been established, the ideal arthroscopic rotator cuff repair method remains controversial [7, 20–22]. In theory, the knotless technique could promote pain relief by avoiding the irritation, strangulation effect and non-physiological load caused by the medial knots [23]. However, the present outcomes demonstrated no statistical difference between the knotted group and the knotless group in postoperative pain relief. The possible explanation is that rotator cuff tears usually coexist with other pathological factors, such as synovitis, adhesive capsulitis, and subacromial impingement syndrome, which lead to shoulder pain together. During an operation, surgeons repaired the rotator cuff tears, removed subacromial osteophytes, and performed debridement of the inflammatory tissue. These concomitant procedures would significantly affect pain-relieving and then mask the subtle differences in the effects of various repair techniques on pain relief [24, 25].

Regarding shoulder dysfunction, adhesive capsulitis and rotator cuff lesion are the most prevalent pathogenesis. Rotator cuff repair can restore the shoulder force couple and release the hyperplastic capsule, which removes the pathological basis of shoulder dysfunction and provide a base for rehabilitation after surgery. Though the knotted suture bridge technique has been proven to have greater strength fixation than the knotless technique, the biomechanical strength of either technique is sufficient for postoperative rehabilitation [26, 27]. In addition, the postoperative rehabilitation programs used in the two groups in the included studies were similar. Therefore, it is reasonable that there were no statistical variation in function scores, and ROM among the two groups.

The efficiency of the two techniques in prevention re-tears is one of the most controversial topics.To achieve healing of tendon to bone, an optimal rotator cuff repair technique should achieve both forceful anatomical reconstruction of the footprint and biological factors (especially adequate blood supply). Although knotted suture bridge techniques could provide superior biomechanical strength of repairs compared with the knotless ones, they could result in a strangulation effect on tendons and then compromise blood flow for healing. A decrease (44.6%) in the blood flow at the repair site of tendons after knotted suture bridge repairs was found by doppler examination [28]. This decline in blood flow was considered to hamper tendon healing process. Furthermore, the strangulation effect of the knotted techniques might induce necrosis of the rotator cuff tendon [20]. Together, these factors might elevate the chance of re-tears or unhealing following rotator cuff repair utilizing knotted suture bridge techniques. The knotless techniques were reported to possess greater self-reinforcement effect, which means that it could also provide reliable strength of the fixation without strangulation effect on tendons [29]. In addition, without medial knots, the knotless suture bridge technique can distribute the tension better, thus avoid tension overload at the repaired tendons [30]. Hence, the knotless techniques were expected to reduce the risk of re-tear in theory. However, our Meta-analysis revealed no statistical difference in re-tear rates among the two procedures, which means that the knotless suture bridge techniques still fail to overcome the faults of the knotted techniques. Fortunately, most re-tears were asymptomatic and had few effects on function [31]. Given that the complication rates of the two techniques were low and most complications were easy to cure, it is reasonable to regard both the techniques with good safety profiles.

This investigation’s primary strength is it provided a multi-dimensional quantitative comparison of clinical outcomes (including pain relief, function, ROM, re-tear, and complications) between the two techniques. However, our investigation has several possible drawbacks. Firstly, most of the selected trials were retrospective and non-randomized, reducing the evidence quality for our conclusions. Secondly, owing to the drawback of the original data in the selected trials, we failed to perform a subgroup analysis of some confounding variables, including tear size and tendon quality, which may lead to omitting some essential conclusions. Third, the number of studies included was small, diminishing the trustworthiness of this study’s findings. Consequently, care must be used while interpreting the data and selecting the appropriate procedure.

## Conclusion

For arthroscopic rotator cuff repairs, there were non-statistically differences in medical results among knotted and knotless suture-bridge procedures. Both techniques showed excellent clinical outcomes and could be used in treating rotator cuff tears with reasonable safety. However, further research is required to assess the clinical efficiency of the two techniques for different tear patterns (varying in size, location, and shape of tears, tendon quality, and comorbidity) to provide a basis for individualized treatment.

## Electronic supplementary material

Below is the link to the electronic supplementary material.

Additional File 1: PRISMA 2020 Checklist

Additional File 2: PRISMA 2020 flow diagram for new systematic reviews which included searches of databases and registers only

## Data Availability

All the data generated/analyzed in this study were included in this published article.
