# Supplementary material for: Comparison of clinical outcomes of arthroscopic rotator cuff repair utilizing suture-bridge procedures with or without medial knots: a meta-analysis
Source: BMC Surg. 2023 Jun 13;23:158. doi: 10.1186/s12893-023-02060-0 (PMC10265913; doi:10.1186/s12893-023-02060-0)
Supplement: Supplementary file 2 — Additional File 2: PRISMA 2020 flow diagram for new systematic reviews which included searches of databases and registers only [file 12893_2023_2060_MOESM2_ESM.docx]

**Identification of studies via databases and registers**

Records removed *before screening*:

Duplicate records removed (n = 79 )

Records identified from

Databases：

Pubmed 127

Embase 85

Cochrane 4

(Total n=216)

**Identification**

**Screening**

Records screened

(n = 137 )

Records excluded**

(n = 118 )

Reports not retrieved

(n = 1 )

Reports sought for retrieval

(n = 19 )

Reports excluded:

The data was incomplete (n = 4)

Adoption of special suture techniques (n =3 )

Reports assessed for eligibility

(n = 18 )

**Included**

Studies included in review

(n = 11 )

*Consider, if feasible to do so, reporting the number of records identified from each database or register searched (rather than the total number across all databases/registers).

**If automation tools were used, indicate how many records were excluded by a human and how many were excluded by automation tools.

*From:*  Page MJ, McKenzie JE, Bossuyt PM, Boutron I, Hoffmann TC, Mulrow CD, et al. The PRISMA 2020 statement: an updated guideline for reporting systematic reviews. BMJ 2021;372:n71. doi: 10.1136/bmj.n71

For more information, visit: <http://www.prisma-statement.org/>
